# Functionalization of collagen fiber with nano-islands of silver via atomic layer deposition to promote bone healing

**DOI:** 10.1016/j.heliyon.2025.e42177

**Published:** 2025-01-23

**Authors:** Sarah Hashemi Astaneh, Leonardo P. Faverani, Harshdeep Bhatia, Eduardo Dallazen, Monique Gonçalves Costa, Edilson Ervolino, Valentim A.R. Barão, Cortino Sukotjo, Christos G. Takoudis

**Affiliations:** a Chemical Engineering Department, University of Illinois Chicago, Chicago, IL, 60607, USA; b Department of Diagnosis and Surgery, Sao Paulo State University (UNESP), Araçatuba, São Paulo, 16015-050, Brazil; c Department of Oral Diagnosis, Piracicaba Dental School, Universidade Estadual de Campinas (UNICAMP), Piracicaba, São Paulo, 13414-903, Brazil; d Department of Basic Sciences, Sao Paulo State University (UNESP), Araçatuba, São Paulo, 16015-050, Brazil; e Department of Prosthodontics and Periodontology, Piracicaba Dental School, Universidade Estadual de Campinas (UNICAMP), Piracicaba, São Paulo, 13414-903, Brazil; f Department of Prosthodontics, School of Dental Medicine, University of Pittsburgh, PA, 15213, USA; g Biomedical Engineering Department, University of Illinois Chicago, Chicago, IL, 60607, USA

**Keywords:** Collagen functionalization, Silver nano-islands, Atomic layer deposition, Bone healing

## Abstract

Modern techniques of thin film deposition (e.g., atomic layer deposition [ALD]) have paved the way for the modification of the surface of target substrates with thin films, nanoparticles, or other types of nanomaterials. This novel way can improve the base material's properties and enhance specific properties through adding functionalized groups to the surface. In this study, ALD of silver was conducted on commercially available Type I collagen membrane to improve its bioactivity and promote bone healing. Two different sample groups were studied: pristine collagen and silver-coated collagen via ALD (Ag/Collagen). Chemical and morphological changes of the collagen membrane were investigated with X-ray photoelectron spectroscopy and scanning electron microscopy and the bioactivity of functionalized collagen with silver was studied in vitro and in vivo. Nano-islands of silver were obtained on collagen fibrils with an average diameter of ∼16 nm. Comparison of gingival cells cultured on pristine collagen, and silver-coated collagen, demonstrated that the attained silver nanoparticle size and concentration are below the toxicity level of silver. In vivo assessment in rat model showed the biocompatibility of the Ag/Collagen, and greater new bone formation compared to control. This novel solvent-free method can be used to functionalize sensitive materials used in surgeries as bone grafting agents to enhance osteopromotive properties without any adverse effects to the cellular environment.

## Highlights

• For the first time, collagen (COL) was coated with silver nano-islands, ∼16 nm in size, using atomic layer deposition.
• Coated collagen (COL-1300Ag) did not affect cytotoxicity, confirmed by in-vitro and in-vivo analysis of animal experiments.
• In-vivo experiments revealed adding silver to collagen increased newly formed bone by an order of magnitude compared to COL.
• In-vivo experiments revealed that adding silver to collagen increased the amount of newly formed bone in large defects.

## 1 Introduction

The use of collagen membrane for bone grafting is becoming more common in the bio-implant industry [[1], [2], [3], [4]]. Collagen is a structural protein, and each molecule tends to rearrange its structure into a triple helix to maximize its stability [1]. These structures, known as fibrils, are responsible for the fibrous collagen membrane, giving it a high surface area. The advantages of collagen membranes are its biodegradability and resorbability that don't require a second surgery for membrane removal. Many efforts have been made to functionalized the collagen, like covalent crosslinking of the collagen membrane [1], and surface functionalization using nanomaterials [5]. Furthermore, to enhance osteo-promotive property and biomaterials integration of collagen fiber, different approaches have been used to modify it, ranging from complex hydrogels [[6], [7], [8]], to phosphates of calcium [9], and use of ceramics [5,10,11].

Surface functionalization using thin films of metals or ceramics to enhance performance of organic biomaterials has been demonstrated on collagen [5,10,12] previously. In those studies, Atomic Layer Deposition (ALD) was used to coat different thin films on collagen in a controllable manner, and that lead to enhanced bone regeneration using MgO [5], bioactivity using TiO_2_ [10] and conductivity using Pt [12]. ALD has been established in the semiconductor industry as one of the promising methods for thin film deposition [13,14]. Comparing ALD with other thin film deposition methods (e.g., electroplating, spin coating, sputtering, physical vapor deposition, and chemical vapor deposition) [15], an advantage of ALD is its excellent controllability of thickness of the targeted thin film down to Ångstrom length scale [16]. Another outstanding benefit of ALD is the conformality it offers, even on three-dimensional (3D) structures including carbon and titania nanotubes [[17], [18], [19]], and biomaterials like collagen [20,21]. Another factor to consider when working with collagen is its thermal degradation and resorbability. Therefore, functionalization of collagen using a low temperature vapor-phase deposition technique such as ALD is of great interest to overcome these challenges. Besides collagen, many other organic substrates have already been successfully functionalized using ALD, such as polydimethylsiloxane [22], polymethylmethacrylate [23], cellulose [24,25] and polypropylene [26] due to its low reaction temperature and strong chemical bonding to the substrate.

A review of the current literature targeting materials that would not only help with cell and tissue development but also speed up bone healing was performed [[27], [28], [29], [30], [31], [32], [33]]. Furthermore, studies suggest that surgical areas are susceptible to bacterial infections which can lead to other complications for recovering patients that have a weakened immune system [[34], [35], [36]]. Therefore, silver was considered as a potential candidate, as it is among the best-known antibacterial agents [37,38] which also has been used recently to enhance bone regeneration [27,32,39,40]. The use of silver for promoting bone growth has been demonstrated on many different substrates, all of which showed an enhancement in antibacterial activity without affecting the biological response [32,[41], [42], [43]]. Recently, silver has also been used in conjunction with graphene oxide (GO), to enhance its antibacterial response [[44], [45], [46], [47]].

Methods for incorporating silver have mostly involved impregnation of the substrate with silver nanoparticles(AgNPs) [38,[48], [49], [50]], which has been proven to enhance bone integration since the 1970s [51]. One of the most recent developments in AgNPs depositions are in the field of electrophoretic deposition (EPD). EPD is easy, low cost and despite being a wet process, can provide good thickness control [52,53]. However, as mentioned earlier, collagen's resorbability and thermal degradation make it an unsuitable candidate for any wet processing, unless it was incorporated before or during production of the membrane, which has led to commercially available products like, ColActive Plus Ag (Covalon) and DermaCol Ag (DermaRite) that use Ag^+^ ions in AgCl suspended in a collagen matrix.

Looking at vapor deposition methods for silver, physical vapor evaporation/condensation [49,54], and ALD [21,26,55,56] have been successfully used to deposit silver, out of which only ALD was easily performed at low temperatures, less than 150 °C.

There are studies on plasma enhanced ALD (PEALD) of silver metal with plasma activated hydrogen, or NH_3_ as the reducing agents [[57], [58], [59], [60]]. There are a few reports on thermal ALD of silver on silicon [55], silica gel powder [61] as well as polypropylene [26]; however, due to the narrow working temperature of silver precursors, silver ALD remains challenging. (Triethylphosphine)(6,6,7,7,8,8,8-heptafluoro-2,2-dimethyl-3,5-octanedionate) Silver(I) [Ag(fod)(Pet3)] is the precursor that was reported to work with both PEALD [60] and thermal ALD [26,55].

The objective of the study was to functionalize Type 1 collagen membrane with silver using ALD and test its impact on bone healing and remodeling in Wistar rats. Chemical and morphological characterization was performed and compared the changes before and after silver deposition. Bioactivity of silver-coated collagen is evaluated in vitro by using 3-(4,5-dimethylthiazol-2-yl)-2,5 diphenyltetrazolium bromide (MTT) assays. To our knowledge, this is the first report on coating Type I collagen with ALD silver and studying the biocompatibility of this novel ALD material in vitro and in vivo.

## 2 Materials and methods

Triethylphosphine(6,6,7,7,8,8,8-heptafluoro-2,2-dimethyl-3,5-octanedionate) silver(I) or Ag(fod)(Pet3) from Strem chemicals was used as the silver precursor and it was maintained in a bubbler at 96 °C during all depositions. As received Ag(fod)(Pet3) was kept at a temperature lower than −18 °C; at that temperature Ag(fod)(Pet3) was white crystalline solid, while at liquid state it turned yellowish. Other researchers also reported this change [62]. For silver ALD, silver cation in the Ag(fod)(Pet3) precursor needs to be reduced to its metallic state. Conventional reducing agents like H_2_ and NH_3_ are not reactive enough to reduce silver in thermal ALD and are reported to only work when plasma is involved to activate them [60,62]. Therefore, dimethylamine borane (DMAB) complex ((CH_3_)_2_NH·BH_3_) from Sigma Aldrich was used as a reducing agent to react with Ag(fod)(Pet3) and leave deposited metallic silver on the substrate [21,55,63]. The DMAB bubbler was kept at 52 °C. This reducing co-reagent first introduced by Kalutarage et al. [63] for ALD of thin copper films at low temperature and later it was used by Makela et al. in the deposition of silver thin films via thermal ALD on Si and glass [55]. Here, commercially available absorbable Type 1 collagen membrane sourced from Bovine Achilles tendon (BIOMEND®) distributed by ZIMVIE was used “as received” as the substrate.

Small cut-outs of silicon approx. 1.5 cm (about 0.59 in) X 1.0 cm (about 0.39 in) and 525 ± 25 μm thickness (WAFERPRO, item number: C04007) were used as reference samples for optical thickness measurements. Prior to deposition, these silicon cut-outs were rinsed with deionized water from a Barnstead™ Nanopure Infinity lab water system (catalog id: D8961) and then dried with N_2_ (Praxair NI 4.8-T, purity: 99.998 %).

Thermal ALD of silver was performed in patented, custom-built ALD system, previously described in Refs. [64,65]. The system can conduct ALD or CVD deposition in the same reaction chamber and can switch between the deposition modes. The system can deposit multi-metal films and multi-layer films of alternating ALD and CVD films. This flow-type reactor has two stainless steel precursor delivery lines and a separate line for oxidizer delivery. This design makes the system flexible for depositions with either precursor/oxidizer or precursor/co-reactant. For silver ALD, one of the bubblers was filled with silver precursor and the other one with the DMAB complex. The precursor and the reducing agent were sequentially introduced into the reactor using a custom-written LabView computer program controlling the open/close sequencing of pneumatic Swagelok valves. Both precursor and co-reactant pulse/purge times were optimally chosen at 4 s/6 s and 3 s/20 s, respectively. One limitation with the silver precursor is its narrow working temperature. Its degradation temperature was reported as 140 °C, while its evaporation temperature is 91–106 °C [55,62]. This narrow temperature range imposes limitation on the ALD processing temperature. In this study, delivery lines in between bubblers and reactor were set at 120 °C and depositions were done at 120 °C. Base pressure and operating pressure of the system were ∼9 and 900 mtorr, respectively.

### 2.1 Surface characterization

Spectral Ellipsometry (J. A. Woollam Co., Model: M − 44) was used to measure the film thickness of Ag on corresponding Si wafer sample, which was loaded as a control sample in each ALD reaction. The chemical composition of the deposited silver was analyzed using Kratos AXIS-165, Kratos Analytical Ltd., UK XPS system equipped with monochromatic Al Kα X-ray source on large area mode (1150 μm × 700 μm). Samples were attached to the sample holder using double-sided carbon tape. During XPS, pressure of the chamber was maintained at less than $10^{-8}$ torr. Casa XPS software was used for peak analysis. Surface morphology was analyzed using high resolution field emission scanning electron microscopy (FESEM) (JOEL JSM-6320F, JEOL, Inc.). Prior to FESEM, pristine collagen and silver-coated collagen samples were sputter coated with 6 nm PtPd to make it conductive for the subsequent FESEM.

### 2.2 Biocompatibility assay

Human gingival fibroblast (HGFs), kindly provided by Dr. Luisa DiPietro at University of Illinois Chicago, College of Dentistry, was cultured via assays employing MTT assay to study the potential cytotoxicity and cell compatibility of silver coated samples to be compared to the pristine collagen membrane. The cells were cultured in Dulbecco's modified Eagle's medium-low glucose supplemented with 10 % FBS and 1 % penicillin streptomycin in a humidified atmosphere at 37 °C and 5 % CO_2_. Cells between passage 2 and passage 5 were used for the experiments. HGFs were cultured at density of 2 × 10^4^/well on top of pristine collagen and silver coated collagen in 24-well plates at a density for MTT study. The cell metabolic activity of the surfaces was determined by the MTT [3-(4,5-dimethylthiazol-2-yl)-2,5-diphenyltetrazolium bromide] assay after day 2 and day 7. All experiments were repeated at least three times. Briefly, cells were incubated with the agents for 4 h. After removing the media and washing each well 3 times with PBS, the cells were further incubated with 100 μL of MTT solution at 37 °C for 4 h. The resulting formazan was solubilized with DMSO, and the absorption was measured at 570 nm in an ELISA reader (DYNEX MRX, USA) Comparing data of control collagen with functionalized collagens is representative of the compatibility of cells with the new functionalized surface.

### 2.3 In vivo tests

This study was approved by the ethical committee in the use of animals from the São Paulo State University (UNESP), School of Dentistry, Aracatuba – São Paulo, Brazil (Protocol #0319/2021), which followed the ARRIVE guidelines in animal studies [66]. Twenty-seven Wistar rats (Rattus norvegicus) ranging from 250 to 300 g (about 10.58 oz) of weight, male, adults (six months of age) were selected for this study. All animals were kept at the Vivarium of the School of Dentistry of Aracatuba (UNESP), being four animals per cage, with controlled temperature (22 ± 2 °C), light cycle (12 h of light and 12 h of dark) and provided solid food and water *ad libitum*.

*Surgical experimental design and groups*: Twenty-seven animals underwent the creation of a critical-size bilateral calvaria defect and were randomized into three groups: 1) BC - clot (negative control group; n = 6 defects): bone defects were naturally filled by blood clot; 2) Collagen Membrane – COL (positive control group; n = 6 defects): bone defects were covered by a collagen membrane; 3) Collagen Membrane with ALD of 1300 Ag cycles – COL Ag1300; n = 6 defects. Each animal had two bone defects created on the calvaria, of which 9 animals from three experimental groups were selected by randomization using envelopes, totalizing 54 defects, considering 3 periods of analysis [5].

*Critical size defect creation*: The animals were sedated with intramuscular ketamine (50 mg/kg) and xylazine (5 mg/kg), and trichotomy was performed in the calvaria region. Local antisepsis was performed with Polyvinyl Pyrrolidone Iodine. A V-shaped incision was performed, and the flap was displaced. A 5 mm (about 0.2 in) bone defect was created on each side of the parietal bone, maintaining the integrity of the dura mater and sagittal sinus [5,67].

Bone defects were covered following the experimental groups previously described (BC, COL and COL-Ag1300), followed by the suture of the flap using Nylon 4.0 (Mononylon, Ethicon, Johnson Prod., São José dos Campos, Brazil). The animals received a single dose of 0.2 mL of Pentabiotic® - Intramuscular (Pentabiotic Veterinário Pequeno Porte, Fort Dodge Saúde Animal Ltda., Campinas, SP). They were euthanized at 7-, 14-, and 28 days after surgery through sedation as performed in the surgery, followed by cardiac perfusion using 800 mL of paraformaldehyde. The samples referring to 7- and 14 days were analyzed for histology/histometric assessment, and at 28 days, they were analyzed for tridimensional microtomography (μ–CT) images and histology/histometric assessment [67].

*Lab processing*: After euthanasia and all the samples collected (calvaria) were maintained in formol for 48 h, followed by all of lab steps to include in paraffin (decalcification in Ethylenediaminetetraacetic acid (EDTA) for eight weeks, diaphanization in Xylol, and inclusion in paraffin) [5,67,68]. The histological sections were obtained with sample cutting in a microtome in slices 5 μm-thick; half of the slides were stained by hematoxylin and eosin (histological/histometric analysis), while the other half were subjected to immunohistochemistry reactions [68,69].

*Computed Microtomographic analysis (μ–CT)*: The samples obtained at 28-days, before lab processing, were scanned by a SkyScan microtomography (SkyScan 1176 Bruker MicroCT, Aatselaar, Belgium, 2003), using the parameters listed following: 8-μm sections, 90 Kv, 111 μA, with copper and aluminum filters and a 0.05-mm rotation pitch. The images were reconstituted with NRecon software (SkyScan, 2011; Version 1.6.6.0), and the image reconstruction and position were performed in the Data Viewer software (SkyScan, Version 1.4.4 64-bit). 3D images were obtained using CT-Vox software (SkyScan, 2012 Bruker MicroCT, Version 1.12.4.0) [67,70].

*Inflammatory profile analysis*: All slides stained with hematoxylin and eosin were first assessed for the inflammatory cells count, emphasizing mononuclear cells and blood vessels. The slides were photographed by a light microscope (DM 4000B, Leica, Wetzlar, Germany) and the software ImageJ (National Institutes of Health, Bethesda, MD, USA) to quantify the data [5,67]. An objective of ×100 magnification was used, and a grid with 130 points was applied to allow cell counting [5,67].

*New bone formation*: The same slides were used for this analysis, and the photomicrography in an ×6.3 magnification (DM 4000B, Leica) was taken. On average, 15 pictures were taken for each defect, and then those images were transferred to Adobe Photoshop CC 2019 for merging into a panoramic view of the bone defect. Using the tool "free hands'' in the ImageJ software, the bone-formed area was measured between the bone stumps [67,70].

*Residual membrane area*: The images, also in a panoramic view, were analyzed in the ImageJ program, using the “free hands” tool to quantify the area of residual membrane in pixels^2^ [5].

*Residual linear defect*: The images in the Image J program were analyzed with the “straight” tool to measure residual linear defects during the times. The amount of closure defect was calculated linearly [5,67].

*Immunohistochemical analysis*: For the immunohistochemical analysis, antigen retrieval was performed by immersing histological slides in a buffered solution (Diva Decloaker; Biocare Medical) in a pressurized chamber (Decloaking Chamber; Biocare Medical) at 95 °C for 10 min. The slides were washed with 0.1 M phosphate-buffered saline (pH 7.4) at each step of the subsequent reaction. To block endogenous peroxidase activity, sections were immersed in 3 % hydrogen peroxide for 1 h and 1 % bovine serum albumin for 12 h. The indirect immunoperoxidase technique was employed using the following primary antibodies: mouse-generated anti-VEGF (SC-7269, Santa Cruz Biotechnology®) [71], and goat-generated mouse anti-OCN (SC-18319, Santa Cruz Biotechnology®) [72]. Histological sections were incubated with secondary antibody for 2 h and treated with streptavidin-peroxidase conjugate for 1 h (Dako Laboratories). The reaction was developed using the chromogen 3,3′-diaminobenzidine tetrahydrochloride (Dako Laboratories) and counterstained with Harris' hematoxylin. The total number of stained cells in each histological section was determined through quantification using the cell counting tool and a grid with 130 points in the ImageJ software (National Institutes of Health, Bethesda, MD, USA) [71,72].

### 2.4 Statistical analysis

The homogeneity of the data was assessed by the Shapiro-Wilk test, showing P > 0.05 for all parameters. The Two-way ANOVA test was applied for cell counting, new bone formed and residual membrane area, considering two sources of variation: experimental groups (BC, COL, and COL-Ag1300) and the period of analysis (7, 14, and 28 days). The One-way ANOVA test was applied for residual linear defect, considering one source of variation: experimental group only on the 28 days. In all tests when P < 0.05, the Tukey post-test was applied. The Two-way ANOVA test was assessed to analyze the number of labeling Osteocalcin (OCN) and Vascular Endothelial Growth Factor (VEGF), followed by the Tukey post hoc test, considering two times for VEGF (day 7 and 14), and for OCN (day 14 and 28).

## 3 Results and discussion

### 3.1 Characterization of collagen samples

The formation of silver on reference collagen was not in the form of a thin film, corroborated by the complex optical behavior of the films observed on control Si. This behavior, also reported previously, made it particularly challenging to develop an appropriate SE model for silver on silicon measurements [58]. The proposed mechanism for thermal ALD with this precursor is the reduction mechanism. In this reduction reaction, the DMAB complex reacts with Ag(fod)(Pet3) precursor and eliminates the remaining ligands of Ag(fod)(Pet3) during the DMAB pulse yielding metallic silver on the surface. The silver deposition mechanism involves a slow step, which results in a nucleation delay [73]. Once silver nucleates on the surface, the growth of silver (like other noble metals) continues in the form of Volmer-Weber mechanism, in the form of islands, because of the high surface energy [58] between the substrate and silver precursor. This island formation of silver [74] is different from the classical layer by layer ALD growth mechanism reported for metal oxides.

**Fig. 1:**
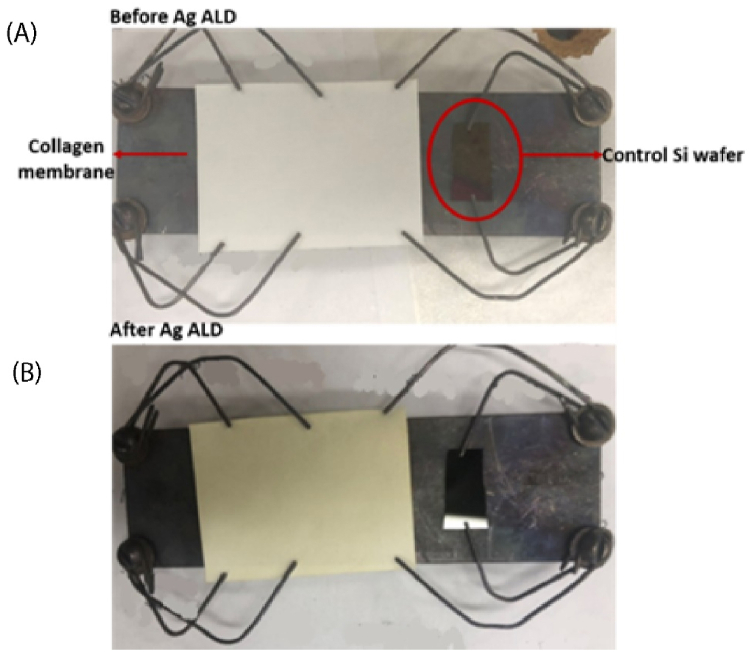
(A) Collagen membrane along with the control Si wafer on the custom-built sample holder, (B) Same samples after ALD process. ALD conditions: 1300 cycles at operation pressure 900 mtorr, precursor pulse/purge time 3 s/5 s and reducing agent pulse/purge time 2 s/10 s, precursor/co-reactant temperature: 96 °C and 52 °C, respectively.

After deposition, however, the collagen substrate showed a notable change in color from white to yellow, as seen in Fig. 1(A) and (B). It has been reported that nano-silver absorbs light at 435 nm, and due to metallic surface plasmons, unaggregated silver nano-islands show yellow color [75,76].

### 3.2 XPS studies

**Fig. 2:**
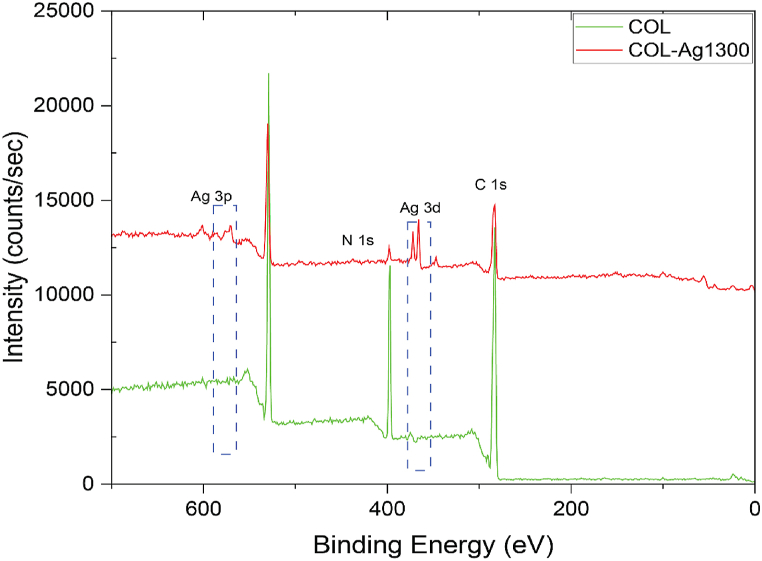
XPS of pristine collagen (COL) and 1300 ALD cycles Ag on collagen (COL-Ag1300) Ag ALD processing conditions: operation pressure 900 mTorr, precursor pulse/purge time 3 s/5 s and reducing agent pulse/purge time 2 s/10 s, precursor/co-reactant temperature: 96 °C and 52 °C, respectively.

XPS is one of the characterization methods for studying the surface chemical composition of samples down to 7–10 nm depth. Fig. 2 shows XPS spectra of pristine collagen (COL) and silver-coated collagen samples after 1300 cycles of the ALD reaction (COL-1300Ag). Samples with ALD silver showed no impurities from the ligands of Ag(fod)(Pet3) (Fluorine, Phosphorus) and the DMAB complex reducing agent (Boron, Nitrogen), suggesting that the reduction reaction reached completion and all Ag^+^ cation of the precursor could be successfully deposited on the surface without being attached to the unreacted side ligands of Ag(fod)(Pet3) [74]. The elemental composition of pristine and silver-coated samples is presented in Fig. 2. After silver treatment, the sample showed characteristic Ag 3p1/2, Ag 3p3/2 peaks at 601 and 571 eV, respectively, and Ag 3d_3/2_ and Ag 3d_5/2_ at ∼373 and ∼367 eV, respectively. These peaks agree with silver ALD features reported in the literature using the same or different precursor/co-reactant systems on different substrates, i.e., silicon and glass [55,77]. Since growth of silver is in the form of nano-islands, even after 1300 cycles, the chemical fingerprint of the collagen substrate is expected to be part of the XPS spectra, e.g., N 1s at 398 eV (Fig. 2), which corresponds to nitrogen atoms of the collagen backbone. To conclude an island-like growth mechanism, comparison was made with a previous study published by Bishal et al. [11] In this study, the formation of titania (TiO_2_) was in the form of thin films and XPS showed that after enough cycles, the C and N backbone of collagen disappeared. However, this was not the case here. After 1300 cycles, both peaks of C and N were observed.

### 3.3 SEM studies

**Fig. 3:**
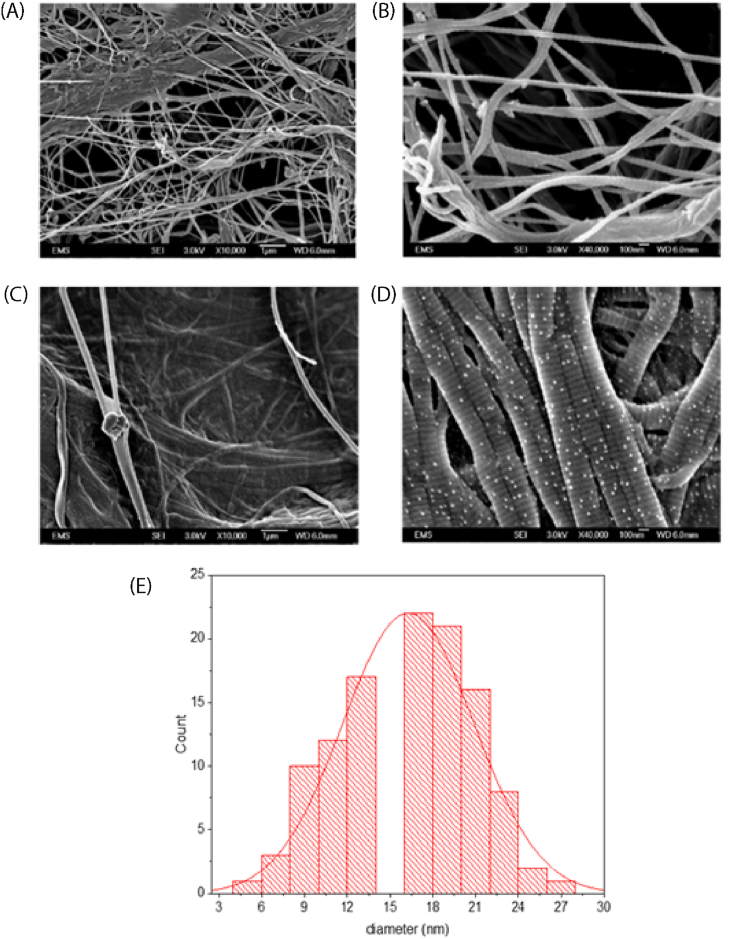
SEM images of the microstructure of A) pristine collagen (COL) at ×10,000 magnification, B) pristine collagen (COL) at ×40,000 magnification, C) Ag coated collagen (COL-Ag1300) at ×10,000 magnification, and D) Ag-coated collagen (COL-Ag1300) at ×40,000 magnification. ALD processing conditions: 1300 cycles at operation pressure 900 mtorr, precursor pulse/purge time 3 s/5 s and reducing agent pulse/purge time 2 s/10 s, precursor/co-reactant temperature: 96 °C and 52 °C, respectively. E) Statistical particle size distribution of silver.

Surface morphology of the pristine collagen and silver-coated collagen samples was studied using FESEM (Fig. 3). Free image processing software “ImageJ” was used to analyze the SEM images. The main difference between uncoated and coated collagen is the presence of tiny amounts of silver islands on and between collagen fibers. The deposited silver indicated circular shape, uniform distribution, and conformal deposition. Fig. 3 (A) and (C), show the microstructure of the samples at ×10,000 magnification, while Fig. 3 (B) and (D) give a closer look at X40,000 before and after silver deposition. As SEM suggested after 1300 cycles of silver ALD, silver particulates formed on the collagen fibers and this deposition showed conformality which means nanosized silver formed on the surface of the collagen and conformally covered all fibers. In real applications, most of these materials are in contact with biofluids that can flow and wet the top and inside fibers. Therefore, ALD can yield functionalized micro/nanostructure even below the top surface. In our ALD system, upon pulsing of the precursor/reducing agent, molecules of the reactants reached the surface, diffused inwards and reached the fibers located below the top surface. The size distribution of the deposited silver is shown in Fig. 3 (E) with an average size of 16 nm, assuming a round shape and no particle aggregation. The morphology and size distribution of the Ag ALD on collagen in our study is like that in Ref. [61] which was reported for plasma-enhanced Ag ALD on silica gel powder, and to the one in Ref. [50] obtained via a solution method on cotton fabric.

Main challenges in using silver for functionalization of materials is its higher cost compared to other metals and its toxicity at higher concentrations. Our SEM results showed that thermal ALD of silver after 1300 cycles yields uniform and conformal coverage of collagen fibers achieved at a low concentration of silver, without significantly changing the morphology, especially without blocking the pores of collagen membrane, which is a critical characteristic for collagen to enable oxygen exchange, micronutrient transfer, blood perfusion, and cell regeneration [1]. As these nano-islands of silver have high surface to volume ratio, this morphology might be preferred over thin film coverage for applications which need high coverage of silver at low concentration, like antibacterial, catalysis, surface plasmon [78] and improvement of near infrared absorption in solar cells [54,79]. In the following section, cytotoxicity of functionalized collagen with silver is further investigated.

**Fig. 4:**
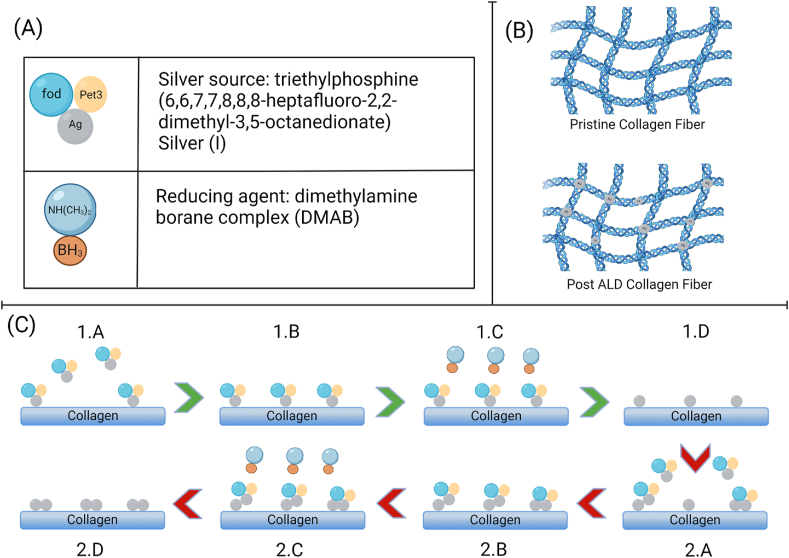
(A) Common names of the silver precursor and reducing agent along with their respective simulated molecule; (B) Schematic of the ALD in the form of metallic nano islands on a collagen fiber mesh; (C) Schematic of Volmer-Weber nucleation and growth mechanism for silver thermal ALD, 1.A Silver precursor pulse. 1.B Chemisorbed silver precursor on the surface. 1.C DMAB complex (as reducing agent of the reaction) pulse. 1.D The reduction reaction between silver precursor and reducing agent. 2.A 2nd cycle of Ag precursor pulse. 2.B Following V-W growth, the new Ag molecule prefers to bind the previously reduced silver metal. 2.C 2nd cycle of reducing agent pulse. 2.D Reducing agent and by products purged after the second round of silver reduction reaction. Illustrations created using Biorender.com.

Based on the results of XPS and SEM the morphology of the silver ALD is in the form of Volmer-Weber mechanism. To understand this mechanism, the two precursors used were visualized as given in Fig. 4 (A). A graphic representation of this form of Ag on collagen after ALD is given in Fig. 4 (B). A schematic of our proposed mechanism is presented in Fig. 4 (C) This supports the results published previously in the case of polypropylene [26] that suggested nano-island growth due to difference in surface energies of the deposited islands and underlying collagen substrate. Despite this difference, the deposited islands were proven to be securely bonded to the substrate, and unaffected by washing or sonicating in DI water. The strength of the chemical bonding is another reason ALD is important to silver deposition on biomedical implants because of silver's cytotoxicity.

### 3.4 Biocompatibility test

**Fig. 5:**
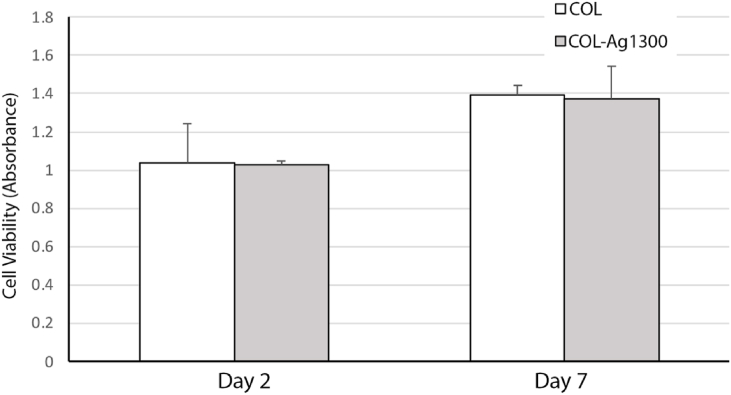
MTT Viability assay results indicating absorbance as a measure of cell viability of gingival cells cultured on the silver-coated samples for day 2 and day 7. COL represents pristine collagen, COL-1300Ag represents 1300 cycles of Ag-ALD on pristine collagen. ALD processing conditions: Deposition temperature 120 °C, operating pressure 900 mTorr, precursor pulse/purge time 3 s/5 s and reducing agent pulse/purge time 2 s/10 s, precursor/co-reactant temperature: 96 °C and 52 °C, respectively.

Although silver is the most preferred commercial antibacterial additive [37], it has been reported that silver can have toxic effect on human cells. Taleghani et al. [80] studied the toxicity of different concentrations of silver nanoparticle solutions on human gingival cells in-vitro. In their study, commercially purchased Plasmachem silver nanoparticles of ∼10 nm were used. Their results showed that toxicity of silver nanoparticles is time and dose dependent; for example, for high concentrations (>10,000 ppb) the viability of cell decreased while lower concentrations did not show significant toxicity. In our work, assays employing 3-(4,5-dimethylthiazol-2-yl)-2,5-diphenyltetrazolium bromide (MTT), gingival cells were cultured as a model cell line to study the potential cytotoxicity of silver-coated samples. The cell culture test was done on a control sample, and 1300 cycles Ag on collagen. In this test, absorbance is expressed as a measure of gingival cell viability. For all tested samples cell proliferation was improved after 7 days. The absorbance values in Fig. 5 indicated that the cell response of silver coated collagens was the same for both day 2 and day 7. That is, our results indicated that the presence of silver nano-islands from ALD had no negative effect on the metabolism of gingival cells, which indirectly represents good proliferation rate of the cells. Hence, this ALD silver is below cytotoxicity levels. This also supports a previous study [26] that found a completely digested 5 mm disk of 1500 cycles of nanoisland-silver coated PP in PBS only contained about 96 ppb of silver, which is significantly lower than toxicity levels reported in the literature [81]. Therefore, due to the established antibacterial nature of silver[50,[82], [83], [84]], membranes coated with silver ALD can be antibacterial and biocompatible.

### 3.5 In vivo analysis

#### 3.5.1 Inflammatory profile analysis

**Fig. 6:**
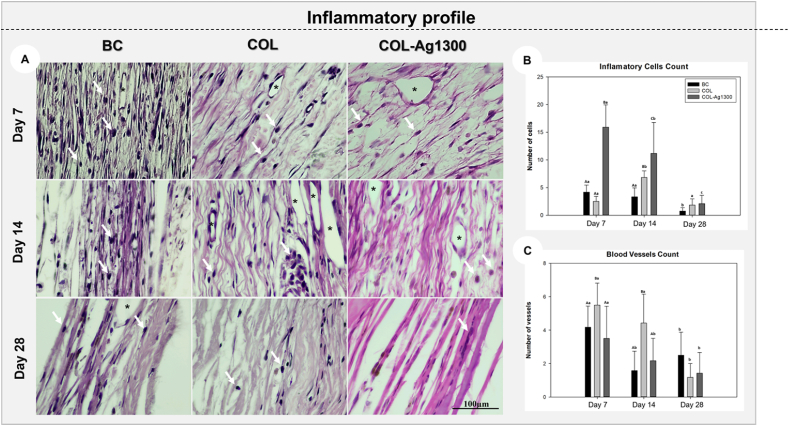
Representative images of the inflammatory response surrounding the tested membranes (A): lymphocytic cells (white arrows) and blood vessels (black asterisk); Mean and standard deviation represented by graphs for inflammatory profile (B) and blood vessels count (C) from experimental groups (BC, COL, and COL-Ag1300) at 7,14 and 28 days postoperatively (HE staining). ∗ Different capital letters represent P < 0.05 in the comparison among experimental groups on each analyzed period. Different lower cases represent P < 0.05 in the intragroup comparison among the periods.

The inflammatory profile of the tested group (COL-Ag1300) did not show any foreign reaction during all analyzed periods. In Fig. 6A, it is clear to notice lymphocytic cells and blood vessels in the entire histological slides on day 7. On days 14 and 28, the organization of collagen fibers showed a great maturation, especially in the COL-Ag1300, compared with control groups. Regarding cell counting, in all analyzed periods, the COL-Ag1300 group showed a higher number of lymphocytes (P < 0.05; Fig. 6B); however, in the intragroup analysis, COL-Ag1300 significantly decreased the number of cells on day 28 compared to other periods (7 and 14 days; p < 0.001). For blood vessels (Fig. 6C), COL showed a higher number of cells on days 7 and 14 (P < 0.05), but all groups were similar at the end of the analysis (day 28; P > 0.05). These results corroborate the data obtained in the in vitro analysis, which showed that due to the antimicrobial properties of silver, membranes coated with ALD silver may have antibacterial properties, and consequently being biocompatible. It is well established in the literature that the clinical applicability of a material applied into an organism is associated with its characteristics of not generating a foreign body reaction and modulating the inflammatory response. It contributes to the angiogenesis process, precursor of the formation of an organized connective tissue [85,86].

Regarding the membrane's biocompatibility, the result of this study is in accordance with [87], which demonstrated that the polymeric biomaterial functionalized with Ag nanoparticles (nAg) assessed was not immunogenic and had better anti-inflammatory activity, especially against secretion of pro-inflammatory cytokines (IL-1β and TNF-α). Thus, indicating that in addition to the known effect of CS, nAg could be responsible for the decrease in the inflammatory response. This study also demonstrated that nAg preserved its anti-inflammatory activity even after incorporation into a biodegradable polymeric biomaterial and could be released to act at the cellular level [87]. In regard to other incorporations to improve osteopromotive action in bone regeneration, *Wachesk* et al. showed that incorporating TiO2 into DLC films significantly increased cell viability compared with membranes with no functionalization. The non-occurrence of any acute inflammatory response, observed with flow cytometry and nitric oxide measurement, shows that DLC and TiO_2_-DLC films were not cytotoxic and the presence of TiO_2_ in DLC films increased the anti-inflammatory response [88].

#### 3.5.2 Histological, immunohistochemical, and computed microtomography (μ–CT) analysis

**Fig. 7:**
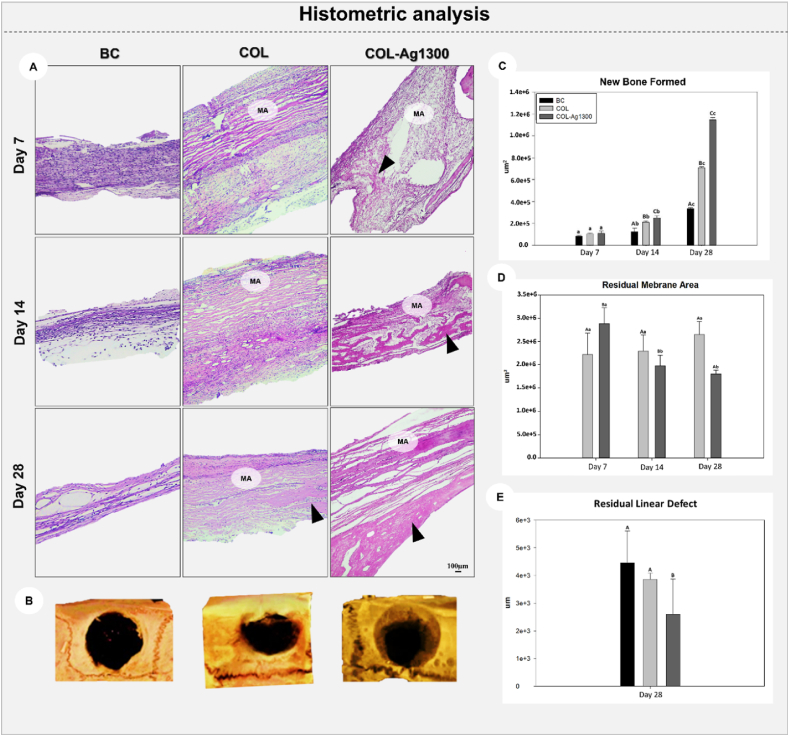
Representative images for the histological and histometric assessment: histological features of membrane behavior on the bone defect (A), Tridimensional images obtained by microtomography scanning (B), Mean and standard deviation represented by graphs for new formed bone (C), residual membrane area (D), and residual linear defect (E) from the experimental groups (BC, COL, and COL-Ag1300) at 7,14 and 28 days postoperatively, which for micro-CT images, only samples on day 28 were assessed. (MA: membrane area; Black triangle: new bone-formed; HE staining). ∗ New bone formed and Residual membrane area: Different capital letters represent P < 0.05 in the comparison among experimental groups on each analyzed period. Different lower cases represent P < 0.05 in the intragroup comparison among the periods. ∗∗ Residual linear defect: Different capital letters represent P < 0.05 in the comparison among experimental groups on day 28 postoperatively.

The chronologic histology of the analyzed groups is represented in Fig. 7A, and the tridimensional μ–CT images are illustrated in Fig. 7B. BC group confirmed that the size of the created defect was critical, showing at the end of the analysis (day 28) that a few amounts of new bone formed were noticed, including close to the bone stumps. The soft tissue was maintained during all analyzed periods. Both membranes (COL and COL-Ag1300) showed integrity until day 28 (MA). COL-Ag1300 showed bone next to the membrane, with trabeculae formation since day 14.

The μ–CT analysis was excellent to analyze the 3D bone regeneration architecture but did not allow the evaluation of the membrane degradation and the cellular composition of regenerated tissues. Thus, histological and histometric analyses are necessary to complement the μ–CT, not only in cell evaluation but also in the full morphological evaluation of regeneration. The importance of using collagen-based membranes was shown through μ–CT, histological and immunohistochemical analysis by some authors, indicating bone regeneration improvement using these membranes [2,[89], [90], [91]]. Dubus et al. showed more mineralized tissue formation, through μ–CT, in critical-size defects of rat calvaria when they used functionalized membranes, compared with no membrane use [92]. In this study, μ–CT analysis shows more hyperdense images in the bone defects in test groups (COL and COL-Ag1300). It suggests more bone formation and, smaller residual bone defects, in the COL-Ag1300 group. The antimicrobial potential of membranes controls the inflammation in the environment of regeneration, as seen in the inflammatory profile section.

#### 3.5.3 Bone histometric analysis

On day 14, both membranes (COL and COL-Ag1300) showed a greater bone-formed area in comparison with BC group (P < 0.05; Fig. 7C), and COL-Ag1300 was higher than COL group, with the highest data on day 28 in all interactions (P < 0.05). For the residual membrane area, COL group maintained its values for all the analyzed periods (P > 0.05; Fig. 7 D), whereas COL-Ag1300 decreased its area at 14 days compared with 7 days (P = 0.43) but maintained it until day 28 (P = 0.07).

The linear defect measurement evidenced at 28 days postoperatively the higher values for BC and COL groups (P > 0.05) and the lowest linear defect for COL-Ag1300 (P < 0.01; Fig. 7E). These results showed that functionalization with silver improved osteo-promotive properties of collagen. Although COL-Ag1300 has reduced its area compared with COL, it remained until 28 days, showing that the functionalization was not harmful to the slow degradation until the end of the analysis, an important characteristic for large reconstructions. Our results corroborate with those in Refs. [8,27] which showed that silver nanoparticles help in the regulation, proliferation, and differentiation of mesenchymal stem cells (MSCs) involved in bone regeneration through (i) chemoattraction of mesenchymal stem cells (MSCs) and fibroblasts for the defect, and (ii) induction of MSC proliferation [8,27,40]. Zhang et al. showed in mouse femoral fracture healing that the interposition of scaffolds produced by collagen and silver nanoparticles between the bone stumps significantly increased the amount of bone, producing a new callus and intense expression of TGF-β and BMP, which these are essential growth factors for the bone regeneration [27]. Casillas-Santana et al. investigated silver nanocomposite in the critical-size defect on an animal model. Nanosilver-chitosan was synthesized and filled an 8 mm calvaria defect created in guinea pigs for osteo-regeneration after 4- and 8-week postoperative periods. In both periods, the nanocomposites promoted great bone regeneration and enhanced the antimicrobial response in the in vitro tests [93].

It is important to notice that our study used membranes to cover the critical-size defects on the calvaria of rats for osteo-promotive activity, not as a bone graft, filling the defect. Even so, it was verified that COL-Ag1300 membranes, through the cellular regulation mechanism, allowed a significant amount of bone, leading to almost closure of the defect, which is paramount for clinical applications.

#### 3.5.4 Immunohistochemical analysis

**Fig. 8:**
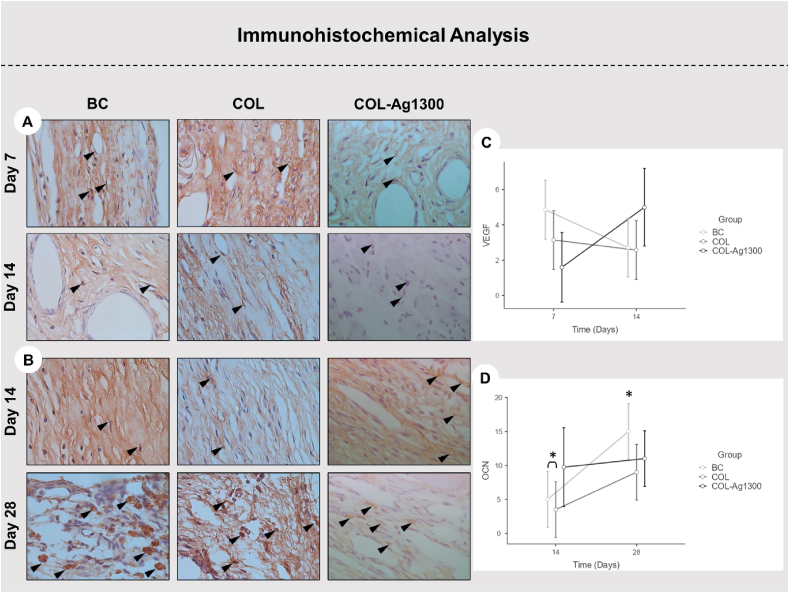
(A) Representative images illustrating endothelial cells' immuno-labeling for VEGF on histological sections on day 7 and 14 (Harris' hematoxylin stain; ×100 objective). Black arrows indicate cells marked for VEGF. (B) Representative images depicting cells immunolabeling for OCN on histological sections on day 14 and 28 (Harris' hematoxylin stain; ×100 objective). Black arrows highlight cells marked for OCN. (C) Line chart presenting the expression of VEGF at 7 and 14 days in the experimental groups. (D) Line chart depicting the expression of OCN at 14 and 28 days in the experimental groups.∗ The black asterisk denotes statistical significance (P < 0.05) in the comparison among experimental groups at each analyzed time point.

The immunohistochemical analysis provided partial support to the μ–CT and histological analyses, corroborating the results reported by Dubus et al. A significant difference (P < 0.05) in OCN values was observed when comparing the results from BC (5.0 ± 3.12) and COL (3.50 ± 2.45) groups at 14 days with BC (15.0 ± 4.34) group at 28 days. Conversely, no difference (P > 0.05) was noted between COL-Ag1300 (9.75 ± 8.66) at 14 days and BC at 28 days. These findings suggest that membranes functionalized with Ag-1300 may contribute to the earlier expression of OCN and subsequent mineralization compared to the control group. However, no significant differences were identified between BC, COL, and COL-Ag1300 groups at 14 days, compared to COL (9.0 ± 2.62) and COL-Ag1300 (11.0 ± 10.20) at 28 days (Fig. 8C and D).

For VEGF labeling, no distinctions were noticed among groups (P > 0.05; Fig. 8A and B). It is crucial to understand that bone repair, even in a size-critical defect, initiates an increase in the number of inflammatory cells as a response to surgical trauma. The neoangiogenic cells represent the beginning of this mechanism. The improvements in the osteopromotive properties of membranes after functionalization, as proposed in this study, may be explained by not blocking the invasion of blood vessels. Collagen membranes exhibit "porosity" among their collagen fibers, allowing osteogenic cells to be provided by the soft tissue flap. In this sense, the histometric analysis complements the findings to elucidate whether the treatment group facilitates bone repair.

## 4 Limitations of the study

Even though this study reinforces the antibacterial capabilities of Silver, there are still some limitations to this study that are further discussed.

This study used Type 1 collagen from natural sources with minimal modification. However, there are other studies where synthetic collagen including silver has been manufactured. Another limitation of this study is that ALD can be a very slow reaction, and in metal depositions, ALD cycles numbers are usually high (over 1000) to ensure that a uniform size distribution of metal islands is present on the surface. With cycle times ranging from 30 to 60 s, this process can be time consuming. However, fast ALD in the form of spatial ALD has been realized commercially and there are reactors that can deposit the same amount of material much faster than conventional, temporal ALD [58,[94], [95], [96], [97]].

Regarding in vitro and in vivo assessments, there are inherent challenges in drawing comparisons with clinical applications. Nevertheless, rodents exhibit a high degree of genetic similarity to humans, approximately 95 % [98]. Most biomaterials require pre-clinical evaluations to verify their biocompatibility and potential to enhance bone healing before clinical use.

## 5 Summary and conclusions

Commercially available collagen was functionalized with nano-islands of silver via thermal ALD. Surface characterization of the samples demonstrated that silver was successfully deposited on collagen samples. SEM revealed that silver deposition is in the form of relatively uniform nano-islands with an average size of ∼16 nm. Silver-coated collagen was found to be biologically compatible with gingival cells and they exhibited no negative effect on the biocompatibility of the collagen, and growth and proliferation of cells. Although the membrane area for COL-1300Ag decreased over time, bone formation in these samples was highest as compared to BC and COL. Collagen is a bioresorbable membrane which makes it difficult to get functionalized via methods which involve solvents. Therefore, our proposed solvent-free silver ALD process is novel and favorable to functionalize the surface of such a bioresorbable substrate without any adverse effects.

## CRediT authorship contribution statement

**Sarah Hashemi Astaneh:** Writing – review & editing, Writing – original draft, Validation, Investigation, Data curation, Conceptualization. **Leonardo P. Faverani:** Writing – review & editing, Writing – original draft, Resources, Methodology, Funding acquisition, Formal analysis, Data curation, Conceptualization. **Harshdeep Bhatia:** Writing – review & editing, Writing – original draft, Investigation, Formal analysis, Data curation, Conceptualization. **Eduardo Dallazen:** Writing – original draft, Resources, Investigation, Formal analysis, Data curation. **Monique Gonçalves Costa:** Resources, Methodology, Investigation, Formal analysis, Data curation. **Edilson Ervolino:** Resources, Investigation, Formal analysis, Data curation. **Valentim A.R. Barão:** Resources, Methodology, Investigation, Data curation. **Cortino Sukotjo:** Writing – review & editing, Validation, Supervision, Project administration, Methodology, Formal analysis, Conceptualization. **Christos G. Takoudis:** Writing – review & editing, Supervision, Project administration, Investigation, Funding acquisition, Conceptualization.

## Data availability statement

Data will be made available on request. For requesting data, please write to the corresponding authors.

## Ethics declaration statement

The authors confirm that the study follows Elsevier's Publishing Ethics Policy. The in-vivo studies reported in this study follow the ARRIVE 2.0 Guidelines (Animal Research: Reporting of In-Vivo Experiments).

## Declaration of competing interest

The authors declare the following financial interests/personal relationships which may be considered as potential competing interests: Valentim Barao reports article publishing charges was provided by Cell Press. Co-author Valentim Barao is an Associate Editor of Heliyon If there are other authors, they declare that they have no known competing financial interests or personal relationships that could have appeared to influence the work reported in this paper.
